# Exploring the association of Bone Alkaline Phosphatases And Hearing Loss

**DOI:** 10.1038/s41598-020-60979-3

**Published:** 2020-03-04

**Authors:** Zhu Wei Lim, Wei-Liang Chen

**Affiliations:** 1 Division of Family Medicine, Department of Family and Community Medicine, Tri-Service General Hospital; and School of Medicine, National Defense Medical Center, Taipei, Taiwan, Republic of China; 2 Division of Geriatric Medicine, Department of Family and Community Medicine, Tri-Service General Hospital; and School of Medicine, National Defense Medical Center, Taipei, Taiwan, Republic of China; 3 Department of Obstetrics and Gynecology, Ditmanson Medical Foundation Chia-Yi Christian Hospital, Chiayi, Taiwan

**Keywords:** Biomarkers, Disease prevention

## Abstract

Hearing loss becomes increasingly common with age and affects quality of life. Recently, scientists have published articles about the relationship between metabolic disease and hearing loss. Metabolic disease was previously found to be highly related to an increase in alkaline phosphatase. Thus, there may be an indirect relationship between alkaline phosphatase (ALP) and hearing loss. In this paper, we will demonstrate the relationship between ALP and hearing loss. We included 3877 National Health and Nutrition Examination Survey (NHANES) participants, who represent the noninstitutionalized civilian population in the United States from age 20 to age 69, and examined the association between ALP and frequency distributions of pure-tone air-condition (PTAC) thresholds. After adjusting for pertinent variables, the subjects who belonged to the higher quartiles of ALP tended to have worse hearing thresholds (pure tone average at high and low frequencies) than the first quartile of ALP (p < 0.001). The results showed a positive correlation between ALP and hearing loss, in both males and females (p < 0.001) and in subjects whose body mass indices (BMI) were less than 30 (p < 0.001). In conclusion, ALP may play a role in detecting hearing loss.

## Introduction

The United States government has paid more attention to auditory healthcare recently due to the prevalence of adult hearing loss between ages 20 and 69. Approximately 37.5 million of American adults aged 18 and over have suffered hearing loss in the national health interview survey in year 2012^1^. About 28.8 million of them could benefit from wearing hearing devices^2,3^. However, only approximately 16 percent of adult aged 20–69 have ever used the hearing devices, based on the manufacturers’ voluntary reports of registered devices to the U.S. Food and Drug Administration, 2012. Therefore, strategy to postpone the onset of hearing loss is needed in order to improve quality of life and reduce medical expenses^4^. There are a myriad of intrinsic and extrinsic factors concerning hearing loss issues, such as age^5,6^, gene polymorphism^5,6^, noise and digital music exposure^7^, poor nutrition and lifestyle^6^, current ototoxic drugs used^5,6^, and secondhand smoke^8^. In addition to these factors, over 50% of patients with hearing impairment have previously unrecognized underlying diseases, such as hypertension^9^, diabetes mellitus (DM)^10^, metabolic syndrome^11^, osteoporosis^12,13^ and Paget disease^14^.

Osteoporosis is a metabolic disease that raises alkaline phosphatase (ALP) levels. ALP, a homodimeric enzyme of 86 kilodaltons, plays a vital role in metabolism within the liver and bone. Clinicians use bisphosphonates to reduce the level of ALP, thus preventing a further decrease in bone mineral density. In addition, bisphosphonates also lower the level of ALP in Paget disease^15^. Recently, researchers have noticed asymptomatic elevated ALP values in normal individuals^14^. In patients with chronic kidney disease, elevated serum ALP levels affect the inflammatory responses and are directly associated with erythropoietin stimulating agent-resistant anemia^16^. A chronically elevated ALP level in a normal person may imply presence of a disease, including hearing dysfunction.

Due to the lack of literature concerning the relationship between ALP and frequency distributions of pure-tone air-conduction (PTAC) thresholds to date, our study was designed to provide insight into this issue. Therefore, we investigated the association between ALP and hearing loss using the National Health and Nutrition Examination Survey (NHANES) data from the year 1999 to 2004.

## Results

### Characteristics of the study population

**Figure 1:**
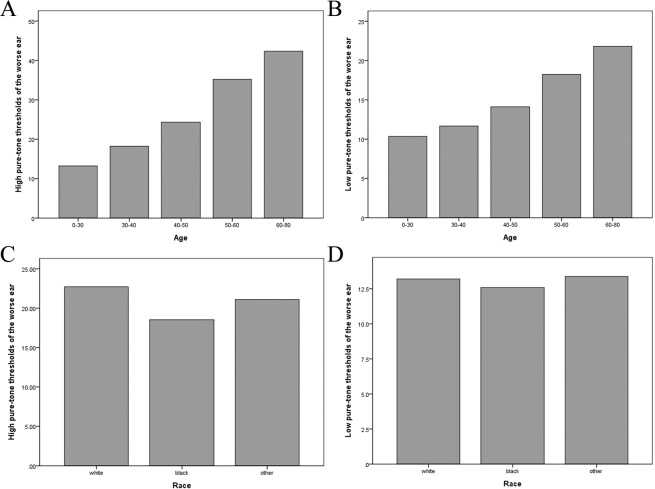
(**A,B**) showed the high and low pure tone thresholds categorized by age. (**C,D**) showed the high and low pure tone thresholds categorized by race.

We included 3877 NHANES participants who represented noninstitutionalized civilian population. The characteristics of the study population are presented in Table 1. Our subjects had a mean age of 39.21 years old (SD = 12.4) and 46.4% of the population was male. Among subjects with higher quartiles of ALP, body mass index (BMI) and uric acid were significantly higher (P < 0.001). DM status was significantly higher in the fourth quartile of ALP (P < 0.001). Figure 1A,B showed aged between 50–80 years old have >25 dB HL (mild hearing loss) in high and low pure-tone thresholds. Figure 1C,D showed non-Hispanic white has >16 dB HL (slight hearing loss) in high pure-tone threshold. Hearing loss continues to be common in older, non-Hispanic whites according to the NHANES data.

**Table 1:** Characteristics of Study Participants.

Characteristics of Study Participants	Quartiles of bone alkaline phosphatase					
	Q1 (<10.6)	Q2(10.6 to<13.8)	Q3 (13.8 to<18.2)	Q4 (>18.2)	Total	*P* value
	(n = 976)	(n = 989)	(n = 967)	(n = 945)	(n = 3877)	
**Continuous variables**^**a**^**, mean ± SD**						
Age (year)	39.48 ± 11.43	39.15 ± 11.53	38.73 ± 12.67	39.49 ± 13.91	39.21 ± 12.41	0.496
BMI (kg/m^2^)	27.18 ± 6.09	28.36 ± 6.13	28.89 ± 6.77	29.06 ± 6.62	28.36 ± 6.45	<0.001
LDL-C (mg/dl)	116.2 ± 34.66	123.76 ± 35.59	123.88 ± 35.85	122.87 ± 35.99	121.60 ± 35.62	0.002
Uric acid (mg/dl)	4.75 ± 1.44	5.19 ± 1.46	5.33 ± 1.41	5.52 ± 1.44	5.20 ± 1.46	<0.001
Worse ear						
Low-PTA (dB)	12.61 ± 9.99	12.99 ± 9.44	13.40 ± 10.53	14.76 ± 12.65	13.43 ± 10.73	<0.001
High-PTA (dB)	20.20 ± 16.70	21.65 ± 17.38	22.48 ± 17.95	24.45 ± 19.20	22.17 ± 17.88	<0.001
Log low-PTA	1.00 ± 0.30	1.02 ± 0.29	1.02 ± 0.30	1.06 ± 0.30	1.03 ± 0.30	<0.001
Log high-PTA	1.19 ± 0.32	1.21 ± 0.34	1.22 ± 0.35	1.27 ± 0.34	1.22 ± 0.34	<0.001
**Categorical variables**^**b**^**(%)**						
Gender(Male)	270(27.2)	464(46.9)	521(53.9)	542(57.4)	1797(46.4)	<0.001
Race						
Non-Hispanic white	558(57.2)	468(47.3)	433(44.8)	336(35.6)	1795(46.3)	<0.001
Non-Hispanic black	199(20.4)	202(20.4)	192(19.9)	189(20.0)	782(20.2)	<0.001
Other	219(22.4)	319(32.3)	342(35.4)	420(44.4)	1300(33.5)	<0.001
Past histories						
Diabetes	42(4.3)	52(5.3)	44(4.6)	79(8.5)	217(5.6)	<0.001
Heart disease	20(2.1)	33(3.4)	27(2.8)	29(3.1)	109(2.8)	0.343
Stroke	11(1.1)	11(1.1)	13(1.3)	19(2.0)	54(1.4)	0.294
Ototoxic medication	167(11.1)	180(9.5)	171(7.2)	190(8.9)	708(9.2)	0.356
Abnormal tympanometry	78(8.7)	94(10.3)	93(10.4)	104(12.2)	369(10.4)	0.121
Abnormal otoscopy	108(17.1)	94(18.2)	70(17.7)	84(20.1)	356(18.3)	0.033

BMI, body mass index; LDL, Low-density lipoprotein;

^a^Values were expressed as mean (standard deviation).

^b^Values in the categorical variables were expressed as %.

### Gender difference in the association of ALP and hearing thresholds

Table 2 shows the positive correlation between ALP and pure tone average at high frequencies (high-PTA) in both men and women. After additional adjustment of models 1, 2 and 3, beta coefficients for ALP and high-PTA were 0.005, 0.005, and 0.005 respectively in men and 0.003, 0.004, and 0.004, respectively, in women (P for trend <0.001). In terms of pure tone average at low frequencies (low-PTA), both men and women showed a positive correlation with ALP. The beta coefficients were 0.004, 0.004, and 0.003 (P for trend <0.001) respectively in men and 0.004, 0.004, and 0.004 (P for trend <0.001) respectively in women.

**Table 2:** Gender difference in the association between alkaline phosphatase with hearing threshold.

Categories	Model^a^ 1 β (95% CI)	*P* Value	Model^a^ 2 β (95% CI)	*P* Value	Model^a^ 3 β (95% CI)	*P* Value
**High PTA**						
Men	0.005 (0.003,0.008)	<0.001	0.005 (0.003,0.008)	<0.001	0.005 (0.002,0.007)	<0.001
Women	0.003 (0.001,0.005)	0.004	0.004 (0.002,0.006)	0.001	0.004 (0.002,0.006)	0.001
**Low PTA**						
Men	0.004 (0.001,0.006)	0.004	0.004 (0.001,0.006)	0.003	0.003 (0.001,0.006)	0.009
Women	0.004 (0.001,0.006)	0.003	0.004 (0.001,0.006)	0.002	0.004 (0.001,0.006)	0.002
**High PTA**						
BMI<30	0.004 (0.005,0.001)	0.007	0.004 (0.001,0.007)	0.006	0.004 (0.001,0.006)	0.011
BMI>30	0.004 (0.000,0.008)	0.050	0.004 (0.000,0.008)	0.038	0.004 (0.000,0.008)	0.039
**Low PTA**						
BMI<30	0.004 (0.002,0.007)	0.002	0.004 (0.002,0.007)	0.002	0.004 (0.001,0.007)	0.004
BMI>30	0.002 (−0.002,0.007)	0.288	0.002 (−0.002,0.006)	0.328	0.002 (−0.002,0.006)	0.384

Model 1 adjust for: age, gender and race.

Model 2 adjust for: age, gender, race, BMI, LDL-C and uric acid.

Model 3 adjust for: age, gender, race, BMI, LDL-C, uric acid, ever told you had a stroke, tympanometry status, ear condition, DM status, present of heart disease and ototoxic status.

### BMI and the association between ALP and hearing thresholds

Table 2 shows a positive correlation between BMI and high-PTA after additional adjustment of three models. Beta coefficients for ALP and high-PTA were 0.004, 0.004, and 0.004 in BMI<30, and in for BMI>30 (P for trend <0.001). In terms of low-PTA, only BMI<30 was related to ALP, and the beta coefficients were 0.004, 0.004, and 0.004 (P for trend <0.001).

### The relationship between different quartiles of ALP and hearing thresholds

We performed a quartile-based analysis by dividing ALP into quartiles, as shown in Table 3. The subjects in the first quartile were set as our reference group. The trends showed significance in these three models regardless of high-PTA or low-PTA. Beta coefficients for Q4 versus Q1 at high-PTA were 0.039 in model 1, −0.040 in model 2 and 0.038 in model 3 (P for trend <0.001). The beta coefficients for ALP comparing Q4 to Q1 at low-PTA were 0.050 in model 1, 0.049 in model 2 and 0.045 in model 3 (P for trend <0.001).

**Table 3:** Regression coefficients of the quartiles of bone alkaline phosphatase with hearing threshold.

Variables		High-PTA		Low-PTA	
		β (95% CI)	P value	β (95% CI)	P value
Model 1	Q2 v.s. Q1	−0.011 (−0.038,0.015)	0.402	0.032 (0.005,0.060)	0.020
	Q3 v.s. Q1	−0.011 (−0.029,0.027)	0.957	0.040 (0.012,0.069)	0.006
	Q4 v.s. Q1	0.039 (0.009,0.069)	0.010	0.050 (0.020,0.080)	0.001
	P for trend	<0.001		<0.001	
Model 2	Q2 v.s. Q1	−0.011 (−0.036,0.016)	0.410	0.031 (0.003,0.058)	0.029
	Q3 v.s. Q1	−0.001 (−0.028,0.028)	0.997	0.038 (0.009,0.067)	0.010
	Q4 v.s. Q1	−0.040 (0.011,0.070)	0.008	0.049 (0.019,0.079)	0.002
	P for trend	<0.001		<0.001	
Model 3	Q2 v.s. Q1	−0.009 (−0.036,0.018)	0.524	0.032 (0.004,0.059)	0.024
	Q3 v.s. Q1	0.001 (−0.027,0.029)	0.941	0.038 (0.009,0.067)	0.010
	Q4 v.s. Q1	0.038 (0.008,0.068)	0.012	0.045 (0.004,0.075)	0.004
	P for trend	<0.001		<0.001	

Model 1 adjust for: age, gender and race.

Model 2 adjust for: age, gender, race, BMI, LDL-C and uric acid.

Model 3 adjust for: age, gender, race, BMI, LDL-C, uric acid, ever told you had a stroke, tympanometry status, ear condition, DM status, present of heart disease and ototoxic status.

Definition of abbreviations: ALP= bone alkaline phosphatase, BMI=Body Mass Index (kg/m^2^), LDL-C = low-density lipoprotein cholesterol, Low-PTA = Pure tone average at low frequencies, High-PTA= Pure tone average at high frequencies.

## Discussion

By evaluating representative data for the US population, we discovered the fourth quartile of ALP was highly related to high-PTA when we weighed the differences between the first, second and third quartiles’ ALP levels. This connection was found not only in obese (BMI>30) individuals but also in underweight, normal weight, and overweight (BMI<30) individuals.

Our results showed a worse hearing threshold in low-PTA was not only observed in women with BMI less than 30 but also in men. As far as we know, women who have low body mass indices are at risk for osteoporosis^17^. ALP has been found at higher levels in osteoporotic postmenopausal women due to high bone turnover rate^12^. In addition, high bone turnover states are known to raise plasma lead levels. A high turnover rates in women who had a higher bone lead level in Q2 and Q3 of ALP compared to Q1 in peri- and postmenopausal women^18^. Therefore, the association between high ALP and high BLL in postmenopausal women in their study corresponds to a worse hearing threshold of low-PTA in low BMI women in our study.

In recent years, scientists have recognized osteoporosis in men. Men suffer from osteoporotic fractures and have twice the 1-year fatality rate of women due to decreased lower body strength^19^. In addition, osteoporotic men trended to have lower free testosterone, higher levels of ALP and sex hormone-binding globulin than osteopenic and normal bone mineral density groups. This finding also supports the correlation between hearing loss and ALP in men, although the association is weak^20^.

Age-related hearing loss is one of the most common conditions affecting older adults. However, hearing loss is also found in younger people without evidence of hair cell loss or absence of auditory nerve synapses in their temporal bones^7,21^. The mechanism is related to the intense metabolic activity such as infection and inflammation, which drive the formation of free radicals and inflammatory cytokines, in both healthy and disease^22–25^. The elevated serum ALP is associated with C-reactive protein (CRP), which indicates ALP level may be a marker of low-grade chronic inflammation^26^. Chronic TNF-alpha release that enters the inner ear may cause bone conduction threshold elevation at the high frequencies^27^. In addition, otitis media-related inflammation also increased air conduction and bone conduction thresholds during childhood and adults stages^28^. While bacterial infection, eosinophilic inflammation, and chronic otitis media deteriorated bone conduction threshold^29^. The increases in stiffness and in mass of the ossicular chain affect bone conduction threshold shifts, which are less likely to accompany aging than are changes in air conduction thresholds^30^. The temporal bone houses the structures of the auditory system^31^. The malleus, incus, and stapes bones are the ossicles, which function to mechanically convert the vibrations into the inner ear. While ALP activity was detected in the ganglion cells and near the temporal bone^31^. Therefore, temporal bone and the ossicles, which contain the ALP activity in the ear, may take part in bone conduction threshold shift^32^.

The mechanism of hearing loss in regard to the increment of ALP remains an uncertainty. To date, ALP with reactive oxygen species (ROS), inflammation, and insulin resistance are possible mechanisms of hearing loss, subsequent to the disturbance of microcirculation in the cochlea. Scientist have established the role of the ROS signaling pathway in osteoblast (OB) differentiation. They demonstrated that the activation of NADPH oxidase was the key reaction to initiate ALP expression. The ROS system therefore produces oxidative stress and induces mitochondrial morphological transition^33^. In addition, vascular calcification raised ALP concentration asymptomatically. A statistically significant correlation between insulin resistance and serum bone ALP levels in vascular calcification, and ALP can be a predictor of cardiovascular events and mortality in patients with DM^34^. Moreover, ALP is capable of dephosphorylating nucleotide phosphatase and lipopolysaccharide to reduce inflammation and coagulopathy, as it shares a similar amino acid sequence with von Willebrand factor and collagen^35^. The positive correlation between inflammation and ALP level mostly presents in healthy elderly people with unrecognized Paget’s disease^5^, osteoporosis^12,13,17^, and presbycusis^5^. The sensorineural hearing loss in Paget disease includes a variety of cochlear lesions^36^ and inferior cochlear vein occlusion that lead to pathologic changes in the stria vascularis and spiral ligament^37^. Based on the findings of Nomura *et al*., ALP shows strong activity in spiral ligament and stria vascularis, and the microcirculatory disturbance in the stria prominence, causes the impaired function in hearing^38^. As previously mentioned, the increased in ALP was parallel to the hearing loss due to the evidence of ALP activity in the temporal bone and cochlea.

Our study had a few limitations. First, the cross-sectional study design with data captured from a single point in time for ALP and hearing loss was a limitation of our study design compared to hearing threshold shift in longitudinal study designs. Second, the self-reported questionnaire of inherited genetic defects and past medical history may have introduced recall bias. Third, the NHANES data only provided the current use of ototoxic medication, instead of hormonal drugs, antidepressants, and anti-inflammatory medicine.

## Conclusion

We concluded that a positive relationship between bone ALP and hearing loss does exist for the general population. For an individual with a BMI<30 and hearing that has remained within the normal range, bone ALP levels seem to be a tool for detecting hearing loss. Because the causal mechanism associated ALP and hearing loss remains unclear, further research should be carried out in order to achieve a better understanding of the underlying pathophysiology of hearing loss.

## Methods

### Ethics statement

The NHANES data were adequately secured and approved by the National Center for Health Statistics Institutional Review Board. Informed consents for all eligible subjects were obtained before the start of a series of surveys with physical and laboratory examination. All methods were performed in accordance with the relevant guidelines and regulations of NHANES.

### Study population

We included 3877 NHANES participants from ages 20 to 69 who represented the noninstitutionalized civilian population in the United States. This population were enrolled from NHANES from the period between 1999 and 2004 and had undergone a series of household interview surveys, physical examinations, and laboratory tests at a mobile examination center (MEC).

### Audiometric measurements

Half of our target populations had undergone audiometric testing randomly after a series of surveys with physical and laboratory tests. Subjects who could not tolerate auditory headphones in MEC sound-isolated rooms were excluded. The test was performed by technicians who had received training from the National Institute for Occupational Safety and Health. They used an AD226 audiometer, TDH-39P headphones and EARTone 3 A earphones for both ears and recorded threshold values from −10 dB to 120 dB. According to the American Speech Language Hearing Association (ASHA), the normal range for hearing thresholds is −10 to 15 dB and the abnormal hearing thresholds (worse ear) is ≥26 dB. The frequency distributions of PTAC thresholds is 0.25–8 Hz^39^. We averaged the thresholds at 3,000, 4,000, 6,000, and 8,000 Hz for the high-PTA while the thresholds at 500, 1,000, and 2,000 Hz were included in the low-PTA, according to NHANES data^40^. In the regression model, we chose the pure-tone thresholds of the worse ear for analysis.

### Covariates

We collected age, sex and race (non-Hispanic black, non-Hispanic white or other) as our demographic data. BMI was calculated as weight divided by the square of the height (kg/m^2^). BMI>30 was referred to as obese. The self-reported questionnaire contained questions about present medical illnesses that had ever been diagnosed by a physician, such as DM, heart disease and stroke, and the current use of ototoxic drugs.

An otoscopic screening examination was used to determine the abnormality of the outer canal of the ear. In addition, tympanometry was abnormal if middle-ear peak pressure showed a value lower than −150 daPa or the compliance was lower than 0.3 ml. The Hitachi analyzer was used to determine biochemical data, such as uric acid and LDL-C. All measurements were standardized according to the guidelines that were recommended by the Centers for Disease Control and Prevention (CDC).

### Statistical analysis

The study used sample weights to account for the complex sampling design and to allow approximations of the United States population, following guidelines of National Center for Health Statistics. The NHANES 1999 to 2004 sample weights adjusted for the differential probabilities of selection and nonresponse in the survey sample. The weight represented the number of individuals in the target population each sample participant was estimated to represent^41,42^. We used the Statistical Package for the Social Sciences version 18.0 to analyze our data. We set two-sided alpha values, with values less than 0.05 as our significant p-values. The dependent variables were the normalized hearing thresholds (high-PTA and low-PTA), which were transformed using the logarithm function. Moreover, we further divided the serum ALP into quartiles as follows: Q1 <10.6 ug/L, 10.6 ug/L ≤ Q2 <13.8 ug/L, 13.8 ug/L ≤Q3 <18.2 ug/L and Q4 ≥ 18.2 ug/L. In the multivariate regression models, we adjusted for age, gender and race for model 1; age, gender, race, BMI, LDL-C and uric acid for model 2; and age, gender, race, BMI, LDL-C, uric acid, stroke, tympanometry status, ear condition, DM status, histories of heart disease, and ototoxic status for model 3.
